# Racial Disparities in 30-Day Outcomes Following Index Admission for COVID-19

**DOI:** 10.3389/fmed.2021.750650

**Published:** 2021-11-02

**Authors:** Vivek Nimgaonkar, Jeffrey C. Thompson, Lauren Pantalone, Tessa Cook, Despina Kontos, Anne Marie McCarthy, Erica L. Carpenter

**Affiliations:** ^1^Perelman School of Medicine, University of Pennsylvania, Philadelphia, PA, United States; ^2^Division of Pulmonary, Allergy, and Critical Care Medicine, Thoracic Oncology Group, Department of Medicine, University of Pennsylvania Perelman School of Medicine, Philadelphia, PA, United States; ^3^Department of Radiology, University of Pennsylvania Perelman School of Medicine, Philadelphia, PA, United States; ^4^Department of Biostatistics, Epidemiology and Informatics, University of Pennsylvania Perelman School of Medicine, Philadelphia, PA, United States; ^5^Division of Hematology-Oncology, Department of Medicine, University of Pennsylvania Perelman School of Medicine, Philadelphia, PA, United States

**Keywords:** readmission, COVID-19, racial disparity, comorbidity, post-acute care

## Abstract

We investigated racial disparities in a 30-day composite outcome of readmission and death among patients admitted across a 5-hospital health system following an index COVID-19 admission. A dataset of 1,174 patients admitted between March 1, 2020 and August 21, 2020 for COVID-19 was retrospectively analyzed for odds of readmission among Black patients compared to all other patients, with sequential adjustment for demographics, index admission characteristics, type of post-acute care, and comorbidities. Tabulated results demonstrated a significantly greater odds of 30-day readmission or death among Black patients (18.0% of Black patients vs. 11.3% of all other patients; Univariate Odds Ratio: 1.71, *p* = 0.002). Sequential adjustment via logistic regression revealed that the odds of 30-day readmission or death were significantly greater among Black patients after adjustment for demographics, index admission characteristics, and type of post-acute care, but not comorbidities. Stratification by type of post-acute care received on discharge revealed that the same disparity in odds of 30-day readmission or death existed among patients discharged home without home services, but not those discharged to home with home services or to a skilled nursing facility or acute rehab facility. Collectively, the findings suggest that weighing comorbidity burdens in post-acute care decisions may be relevant in addressing racial disparities in 30-day outcomes following discharge from an index COVID-19 admission.

## Introduction

Racial disparities in infection, hospitalization, and mortality from coronavirus disease 2019 (COVID-19) have been documented, particularly for Black Americans (1). Recent studies have investigated readmission rates following hospitalization for COVID-19 (2, 3), showing that most readmissions occur within 10 days of discharge (4). There is limited data on the effect of post-acute care on racial disparities in 30-day readmissions. Evaluating determinants of racial disparities in COVID-19 readmissions could enable targeted interventions to address inequities. Thus, we analyzed 30-day outcomes of COVID-19 patients surviving to discharge across a 5-hospital health system.

## Materials and Methods

This retrospective study of 30-day readmission or death among patients hospitalized between 3/1/2020 and 8/21/2020 for COVID-19 was conducted within the University of Pennsylvania Health System and approved by the University's Institutional Review Board. Patients were identified using a dataset of all patients with an order for any COVID-19 test and chest imaging completed within the health system. Only patients admitted within 14 days of placement on the health system's COVID-19 positive registry and with primary diagnosis ICD-10 codes consistent with COVID-19 were included. Patient characteristics from the medical record were compared by race using the chi-squared and Mann-Whitney tests. Logistic regression of 30-day outcome was sequentially adjusted for demographics, index admission characteristics, type of post-acute care, and comorbidities (5). We tested the interaction of race with post-acute care using the likelihood-ratio test and performed stratified regression analyses. All analyses were completed on Stata MP Version 15.1 (StataCorp).

## Results

Of 1,461 admitted COVID-19 patients, 1,174 survived to discharge. 625 patients were Black (53.0%); a majority of the remaining patients were White (*n* = 427, 37.1%), and 10% reported Hispanic ethnicity. Black patients were younger, more likely female, had different forms of insurance, and a greater burden of comorbidities. Index admission length and frequency of mechanical ventilation were similar between Black patients and patients of other races, but the distribution of post-acute care on discharge differed (Table 1).

**Table 1:** Characteristics of Black and Non-Black patients by site of discharge following index admission for COVID-19.

	**Total**	**Black patients**	**All other patients**	*P*-value^*^
*n*	1,174	645	529	
**Racial sub-groups**
Black (%)	645 (54.9)			
White (%)	427 (36.4)			
Other (%)	102 (8.7)			
**Ethnicity**
Hispanic	124 (10.6)	22 (3.4)	102 (19.3)	
**Additional demographics**
Age, median (yrs, IQR)	62 (49, 74)	61 (48, 71)	63 (50, 77)	**0.022**
Female, *n* (%)	566 (48.2)	340 (52.7)	226 (42.7)	**0.001**
**Insurance**
Private (%)	282 (24.0)	159 (24.7)	123 (23.3)	** <0.001**
Medicare (%)	555 (47.3)	290 (45.0)	265 (50.1)	
Medicaid (%)	223 (19.0)	154 (23.9)	69 (13.0)	
Uninsured (%)	38 (3.2)	8 (1.2)	30 (5.7)	
Unknown (%)	76 (6.5)	34 (5.3)	42 (7.9)	
**Comorbidities**
Charlson comorbidity index, mean (SD)	3.2 (3.3)	3.9 (3.6)	2.3 (2.7)	** <0.001**
Type 2 diabetes mellitus (%)	437 (37.2)	289 (44.8)	148 (28.0)	** <0.001**
Chronic obstructive pulmonary disease (%)	124 (10.6)	80 (12.4)	44 (8.3)	**0.023**
Congestive heart failure (%)	243 (20.7)	156 (24.2)	87 (16.4)	**0.001**
Chronic kidney disease (%)	197 (16.8)	147 (22.8)	50 (9.5)	** <0.001**
Hypertension (%)	786 (67.0)	483 (74.9)	303 (57.3)	** <0.001**
Cancer (%)	219 (18.7)	124 (19.2)	95 (18.0)	0.579
History of cerebrovascular infarction (%)	189 (16.1)	128 (19.8)	61 (11.5)	** <0.001**
Sickle cell disease (%)	21 (1.8)	21 (3.3)	0 (0.0)	** <0.001**
**Index admission**
Length of stay (days, IQR)	6 (3, 12)	5 (3, 12)	6 (3, 11)	0.164
Mech. ventilated (%)	152 (12.9)	87 (13.5)	65 (12.3)	0.542
**Post-acute care on discharge**
Home (without services) (%)	523 (44.5)	271 (42.0)	252 (47.6)	** <0.001**
Home health care (%)	352 (30.0)	230 (35.7)	122 (23.1)	
SNF/acute rehab (%)	241 (20.5)	114 (17.7)	127 (24.0)	
Other (%)	58 (4.9)	30 (4.7)	28 (5.3)	
**30-day outcomes**
Readmission (%)	161 (13.7)	105 (16.3)	56 (10.6)	**0.005**
Time to readmission (median days, IQR)	7 (3,14)	6 (3, 15)	7 (3, 13.5)	0.9674
Death at 30 days (%)	20 (1.7)	14 (2.2)	6 (1.1)	0.175
Readmission or death 30 Days (%)	176 (15.0%)	116 (18.0)	60 (11.3)	**0.002**

* *All p-values are for chi-squared or Mann-Whitney tests; chi-squared tests were used for categorical variables and Mann-Whitney tests were used for continuous variables. Bold values denote p-values that were statistically significant at the p < 0.05 level*.

Amongst all discharged patients, 176 patients (15.0%) were readmitted or died within 30 days: 116 Black patients (18.0%) vs. 60 patients of other races (11.3%) (Table 1). A univariate odds ratio of 30-day readmission or death among Black patients was 1.71 (CI: 1.23–2.40, *p* = 0.002) without meaningful change on sequential adjustment for demographics, index admission, and post-acute care (Table 2, Models 1–4). Addition of comorbidities, however, attenuated the odds ratio for race with 30-day readmission or death, and it was no longer statistically significant (Table 2, Model 5).

**Table 2:** Odds ratios of 30-day readmission or death among Black patients vs. all other patients with different post-acute care on discharge after sequential adjustment for other demographics, index admission characteristics, and comorbidities.

	Model 1: univariate^*^		**Model 2: adjusted for age, sex, insurance^†^**		**Model 3: additionally adjusted for index length of stay and ventilation^‡^**		**Model 4: additionally adjusted for discharge site^§^**		**Model 5: additionally adjusted for Charlson comorbidity index^||^**	
	**Odds ratio (95% CI)**	***P*-value**	**Odds ratio (CI)**	***P*-value**	**Odds ratio (CI)**	***P*-value**	**Odds ratio (CI)**	***P*-value**	**Odds ratio (CI)**	***P*-value**
All patients (*n* = 1,174)	1.71 (1.23, 2.40)	**0.002**	1.81 (1.28, 2.56)	**0.001**	1.80 (1.27, 2.55)	**0.001**	1.79 (1.26, 2.55)	**0.001**	1.39 (0.96, 2.01)	0.085
Patients discharged home without services (*n* = 523)	2.12 (1.19, 3.78)	**0.010**	2.09 (1.15, 3.80)	**0.015**	2.06 (1.13, 3.76)	**0.018**			1.74 (0.93, 3.24)	0.082
Patients discharged to home health care (*n* = 352)	1.18 (0.64, 2.17)	0.594	1.31 (0.69, 2.49)	0.417	1.28 (0.67, 2.45)	0.457			0.93 (0.47, 1.86)	0.844
Patients discharged to SNF/acute rehab (*n* = 241)	1.70 (0.87, 3.32)	0.119	2.00 (0.99, 4.06)	0.054	1.91 (0.93, 3.90)	0.076			1.39 (0.64, 3.00)	0.405

*Model Definitions (all regressed on 30 readmission or death, bolded independent variables were significant at the p < 0.05 level in the all patient regression)*.

* *Model 1: ***Black race*** alone (univariate)*.

† *Model 2: ***Black race***, sex, ***patient age***, and ***insurance****.

‡ *Model 3: ***Black race***, sex, patient age, ***insurance***, index admission length of stay (days), index admission mechanical ventilation*.

§ *Model 4: ***Black race***, sex, patient age, ***insurance***, index admission length of stay (days), index admission mechanical ventilation, post-acute care on discharge*.

|| *Model 5: Black race, sex, patient age, ***insurance***, index admission length of stay (days), index admission mechanical ventilation, post-acute care on discharge, ***Charlson Comorbidity*** ***Index****.

When stratified by post-acute care, univariate odds of readmission or death were significantly greater for Black patients discharged to home without services (OR = 2.12, 95% CI: 1.19–3.78, *p* = 0.010) but not to home health care (OR = 1.18, CI: 0.64–2.17, *p* = 0.594) or skilled nursing or acute rehab facilities (OR = 1.70, CI: 0.87–3.32, *p* = 0.119). However, the interaction between Black race and post-acute care was not statistically significant (*p*-interaction = 0.309).

Multiple sensitivity analyses were performed. With 30-day readmission as the sole endpoint and patients who died in the timeframe excluded, the same trend in greater odds of readmission among Black patients was observed on serial adjustment for contributory variables, where odds of readmission were no longer significant after adjustment for comorbidities. Identical trends were observed on sensitivity analyses using eight individual comorbidities instead of the Charlson Comorbidity Index, excluding readmissions to other health systems, and including COVID-19 registry patients with any ICD-10 code.

## Discussion

Black patients were more likely to be readmitted or die at 30 days following admission for COVID-19 across an urban health system after adjusting for demographics, post-acute care, and index admission characteristics. To our knowledge, this is the first study to note a racial disparity in 30-day outcomes following COVID-19 hospitalization. This is consistent with disparities in readmissions seen for conditions that frequently cause hospitalizations, such as myocardial infarctions, decompensated heart failure, and pneumonia (6). For these conditions, disparities in readmissions were also related to the sites of care such that hospitals serving larger minority populations similar to the population in our study had larger disparities in readmission rates. Analysis in 2018 following the institution of Medicare's Hospital Readmission Reduction Program, which penalizes hospitals for higher than expected 30-day readmissions for acute myocardial infarction, heart failure, and pneumonia, suggest that racial disparities have persisted and may have widened for non-targeted conditions at safety net hospitals that care for larger populations of Black patients (7). While the site of care has been associated with racial disparities in readmissions, there are other structural inequities that could contribute to greater readmissions among Black patients. In a national study of readmissions in diabetic patients, other demographic factors, most notably household income, also contributed to the disparity in readmissions along with the site of care (8). We lacked data on household income or other measures of socioeconomic status beyond insurance status.

It should be noted that a greater proportion of the Black patients admitted in our study were females, though sex was not found to be statistically significant in our regressions on 30-day outcomes. The larger proportion of females admitted for COVID-19 among the Black population in our study could reflect a differential impact of the pandemic on the sexes across racial groups. It has been observed that COVID-19 outcomes among males and females can differ between Black and White patients, though previously presented data have described worse outcomes among Black males rather than females (9). Alternatively, the higher proportion of Black females admitted for COVID-19 in our study may be attributable in part to regional population level differences in the ratio of females to males by race above the age of 50 (10), which are due to shorter life expectancy among Black men (11).

Differences in the burden of comorbidities largely explained the racial difference in 30-day outcomes. Greater comorbidities among Black patients have previously been attributed to the effects of structural racism on health and limited access to care (12). Though analyses of racial disparities in readmissions during epidemics and pandemics are limited, there is a robust literature documenting the disproportionate impacts of disease outbreaks on racial minorities, and thorough analysis of the 1918 Influenza Pandemic suggests that structural inequities in access to care contributed to racial disparities in outcomes such as mortality (13). In fact prior to the COVID-19 pandemic, some had predicted that a future pandemic influenza was likely to result in worse outcomes in Black populations due to greater barriers to care and burdens of comorbidities among Black patients (14).

The racial disparity appeared most pronounced among patients discharged home without services. It has previously been noted that readmissions are driven not just by the inpatient care received, but also by the outpatient care and socioeconomic resources available after discharge (15). Post-acute care can range from home services, such as visiting nurses, to care in a facility. Without post-acute care, the effects of structural racism on access to care may be exacerbated. Our study suggests that racial disparities in 30-day outcomes may be most evident in the absence of post-acute care. Paired with the finding that comorbidities explained much of the disparity in 30-day outcomes, these results suggest that weighing comorbidity burdens in post-acute care decisions may help address racial disparities in outcomes of discharged COVID-19 patients.

There are several limitations of this study. First, without information on the reasons for 30-day readmission or death, it is difficult to understand the degree to which 30-day outcomes were driven primarily by COVID-19 versus other conditions. Additionally, this study captured only deaths occurring in the 30-day period after discharge that were recorded in the electronic medical record. Consequently, it is possible that some deaths may have been missed with unclear impacts on the composite 30-day outcome of readmission and death.

Nevertheless, this study has illustrated that Black patients across an urban health system were more likely to be readmitted or die within 30 days after an index admission for COVID-19 in the initial surge of the pandemic. Existing comorbidities appeared to play an important role in explaining racial disparities, particularly among patients without post-acute care. Future studies should explore whether these findings are present in other cohorts, including at different time points in the pandemic. Validation across other cohorts would motivate interventional studies that interrogate the formal incorporation of burdens of comorbidities into the assignment of post-acute care at discharge following hospitalization for COVID-19 as a means of reducing racial disparities in readmissions.

## Data Availability Statement

The datasets presented in this article are not readily available due to the need to maintain patient confidentiality. Requests to access the de-identified datasets should be directed to annemcc@pennmedicine.upenn.edu.

## Author Contributions

All authors listed have made a substantial, direct and intellectual contribution to the work, and approved it for publication.

## Funding

Work on this project was supported with funding from the Penn Center for Precision Medicine.

## Conflict of Interest

The authors declare that the research was conducted in the absence of any commercial or financial relationships that could be construed as a potential conflict of interest.

## Publisher's Note

All claims expressed in this article are solely those of the authors and do not necessarily represent those of their affiliated organizations, or those of the publisher, the editors and the reviewers. Any product that may be evaluated in this article, or claim that may be made by its manufacturer, is not guaranteed or endorsed by the publisher.
